# Identification and Characterization of Copy Number-Associated Driver Genes in Esophageal Squamous Cell Carcinoma

**DOI:** 10.1155/2020/6387519

**Published:** 2020-08-22

**Authors:** Kexin Jiang, Yijiang He, Xu Zhang

**Affiliations:** Radiation Oncology Department of Gastrointestinal Cancer and Lymphoma, Cancer Hospital of China Medical University, Liaoning Cancer Hospital and Institute, China

## Abstract

**Background:**

Esophageal squamous cell carcinoma (ESCC) is a leading malignancy with both high incidence and mortality worldwide. However, the molecular mechanisms of the poor prognosis in ESCC are still unclear.

**Methods:**

We conducted differential expression analysis between ESCC and normal tissues and between ESCC samples with and without CNAs in a given gene. Overrepresentation enrichment and gene set enrichment analyses were used to identify the oncogenic pathways and abnormal transcription factors (TFs). The survival analysis was employed to identify the genes associated with overall survival.

**Results:**

In this study, we aimed to identify and interpret the driver genes triggered by the copy number alterations (CNAs), including CCND1, TEAD4, EIF4EBP1, EGFR, FGFR3, and FZD6. Furthermore, we identified oncogenic pathways, including RTK-RAS, WNT, PI3K, Hippo, and cell cycle, and key TFs including TEAD4, a transcription factor in the Hippo signaling pathway, and LEF1 in the WNT signaling pathway. Furthermore, we observed that upregulations of FGFR3 and EIF4EBP1 were significantly associated with shorter overall survival in ESCC.

**Conclusion:**

In conclusion, the driver genes triggered by CNAs not only exhibited critical functionality but also were clinically relevant in ESCC, which greatly improved our understanding of the molecular mechanisms in ESCC.

## 1. Introduction

Esophageal cancer is the seventh most prevalent malignancy worldwide and ranked sixth in mortality [1] (6.6% of the total cancer deaths). Esophageal cancer mainly consists of two types: esophageal adenocarcinoma (EAC) and esophageal squamous cell carcinoma (ESCC), with ESCC accounting for the majority of esophageal malignancies [2]. ESCC is considered to be associated with environmental risk factors, such as chronic smoking, hot tea drinking, and red meat and alcohol consumption, and previously proposed precursor lesions of ESCC including esophagitis, atrophy, and dysplasia [3, 4]. Though advanced screening techniques have greatly helped the diagnosis of ESCC, it is worth noticing that even for patients with an early-staged ESCC, recurrence or distant metastases shortly after surgery can be observed, which often leads to poor prognosis [2]. In order to develop individualized and targeted therapies for ESCC, molecular mechanism behind ESCC carcinogenesis and metastasis needs to be further clarified.

With next-generation sequencing (NGS) technology, investigation of the genetic background has unveiled the involvement point mutations, insertions, deletions, and copy number alterations/variations (CNA/CNV) in ESCC tumorigenesis. So far, a previous study has demonstrated the interaction between ADH1B and/or ALDH2 risk alleles and environmental risk factor through SNP genotyping in ESCC [5], and another study has identified 26 significantly mutated genes in ESCC, where TENM3 mutations and the TP53 hotspot mutation p.R213∗ were stated to be related to poor prognosis [6]. Notably, nonsynonymous mutation and copy number loss/gain in TP53, KMT2D, NOTCH1, PIK3CA, and FAT1 in ESCC are frequently reported and discussed, as they played important roles in NOTCH signaling, the RTK-MAPK-PI3K pathway, and cell cycle regulation, all of which were closely related to tumor development and differentiation [7–9]. Moreover, a recent study has demonstrated the association between NTRK1 gene amplification and the efficacy of larotrectinib in a metastatic ESCC patient, which hinted that exploring novel CNA/CNV and related driver mutations would provide insights for making therapeutic decisions [10]. In the present study, we conducted a systematic data analysis to identify the driver genes and characterize their functionalities in ESCC, which might provide some evidences for the related research studies.

## 2. Materials and Methods

### 2.1. Data Acquisition

From the UCSC Xena database (http://xena.ucsc.edu/), we downloaded the gene expression data and copy number alterations (CNAs) of ESCC patients, which were collected by the Cancer Genome Atlas (TCGA, https://portal.gdc.cancer.gov) project [11, 12]. The expression levels were normalized at FPKM (fragments per kilobase per million) and log2-transformed. The copy number status of gains or losses and the wild type were determined by their log2 (copy number/2). The gene expression dataset for the independent validation of differential expression was collected from Gene Expression Omnibus (GEO) with accession number GSE45670 [13].

### 2.2. Differential Expression Analysis

The 81 esophageal squamous cell carcinoma (ESCC) and 11 adjacent normal tissues with detailed clinical information from TCGA were used for the differential expression analysis. We applied the Wilcoxon rank-sum test and calculated the fold change to determine if the differences observed were statistically significant, and genes with an adjusted *P* value less than 0.05 and a fold change higher than 2 were considered the differentially expressed genes (DEGs).

### 2.3. Copy Number Alteration-Related Driver Genes

First, we used gene entries from the GENCODE (https://www.gencodegenes.org/) v22 database to annotate the segmented CNAs that we obtained [14]. Utilizing these segmented CNAs, we estimated and assigned values for their corresponding genes, which were calculated as log2 (copy number/2). The expressions of these genes were then taken into consideration. For each of these genes, we measured its differential expression levels between the samples with and without CNAs, and the threshold was set to a *P* value < 0.0001 to identify the driver genes triggered by the corresponding CNAs.

### 2.4. Overrepresentation Enrichment Analysis (ORA)

After identifying these differentially expressed genes between ESCC and normal tissues, we performed an overrepresentation enrichment analysis on the obtained gene set. Enricher function in R *clusterProfiler* package was used to carry out ORA [15], and an adjusted *P* value lower than 0.05 was considered statistically significant [16]. The genes in each oncogenic pathway were obtained from R package maftools.

### 2.5. Cox Proportional Hazard Regression Analysis

We used package *survival* in R to conduct survival analysis. To identify driver genes that could significantly affect overall survival, we performed univariable Cox regression and plotted Kaplan-Meier curves to demonstrate varied survival probabilities among different groups using R package *survminer*.

## 3. Results

### 3.1. Identification of Differentially Expressed Genes between Esophageal Squamous Cell Carcinoma and Adjacent Normal Tissues

**Figure 1:**
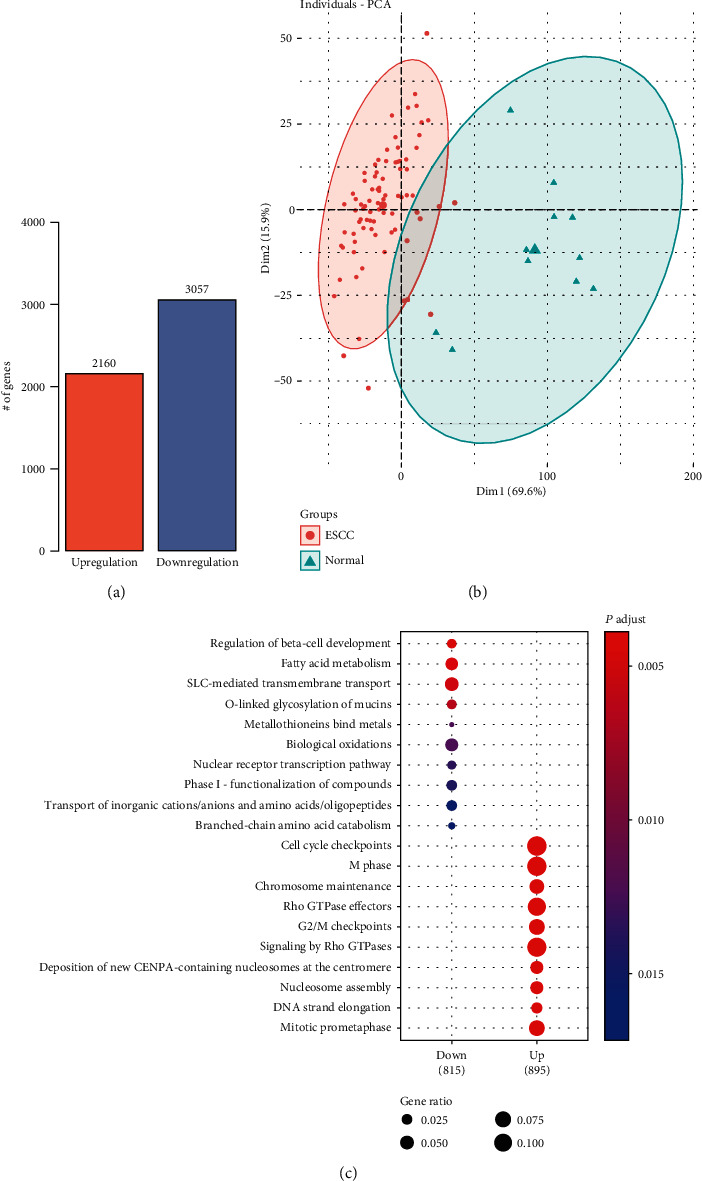
The differentially expressed genes and dysregulated pathways in ESCC. (a) The number of upregulated and downregulated genes in ESCC, which are represented by the orange and blue bars, respectively. (b) The ESCC and adjacent normal samples are represented by the nodes colored by red and green, respectively. The *x*- and *y*-axes represent the top two principal components by principal component analysis (PCA). (c) The pathways enriched by the upregulated and downregulated genes. The node size and color represent the number of genes and statistical significance.

To detect the differentially expressed genes (DEGs) between 81 esophageal squamous cell carcinoma (ESCC) and 11 adjacent normal tissues from the Cancer Genome Atlas (TCGA), we compared the gene expression profiles and identified 2,160 upregulated and 3,057 downregulated genes (Figure 1(a)). The principal component analysis (PCA) of these genes revealed that the ESCC samples could be clearly distinguished from the adjacent normal tissues (Figure 1(b)). Results from functional enrichment analysis on those dysregulated genes hinted that activities of cell cycle-related pathways and metabolism-related pathways were significantly altered in ESCC (Figure 1(c)), respectively. These results suggested that the DEGs could not only differentiate the ESCC from normal tissues but also reveal cancer-related pathways.

### 3.2. Identification of CNA-Triggered Driver Genes

**Figure 2:**
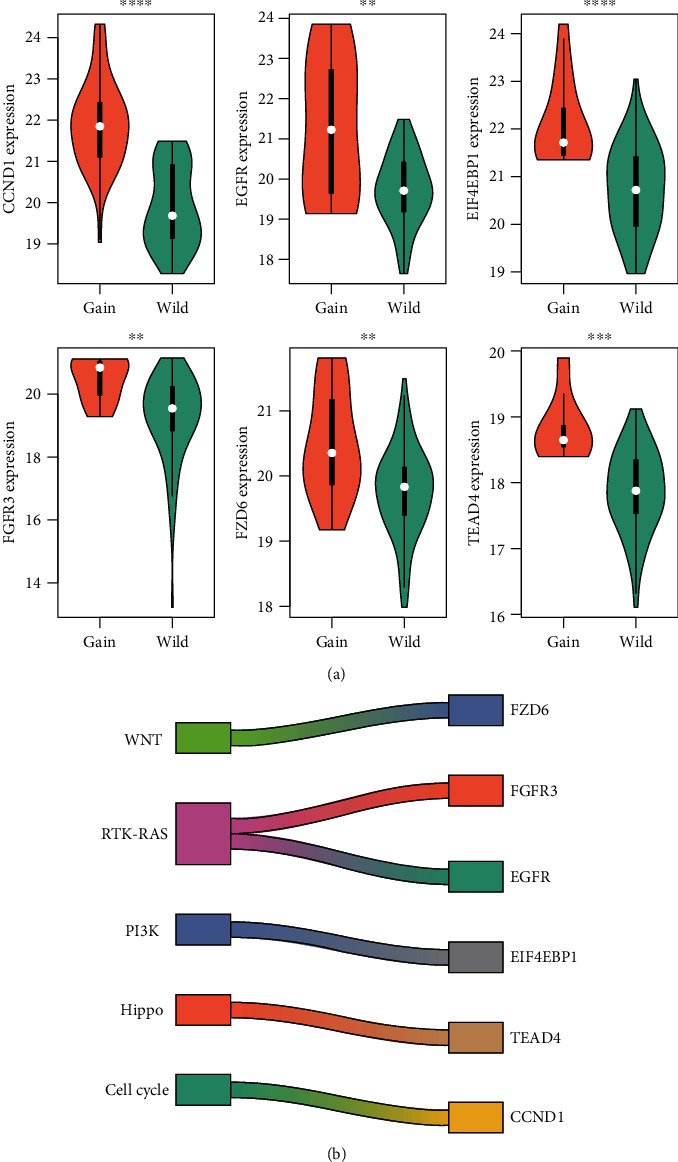
The driver genes and their regulating oncogenic pathways. (a) The expression levels of the CNA-related driver genes between the samples with and without CNAs. (b) The regulating oncogenic pathways that the driver genes were involved in. The symbols of ∗, ∗∗, ∗∗∗, and ∗∗∗∗ indicate the *P* values less than 0.05, 0.01, 0.001, and 0.0001, respectively.

To further detect driver genes triggered by the copy number alterations (CNAs), we examined whether the expression levels of DEGs were triggered by the CNAs. In total, we identified six driver genes including CCND1, TEAD4, EIF4EBP1, EGFR, FGFR3, and FZD6, which were highly upregulated in the samples with gains of the corresponding genes (Figure 2(a), Wilcoxon rank-sum test, *P* < 0.05). Particularly, FGFR3 and EGFR were involved in the RTK-RAS pathway, and the remaining driver genes including FZD6, EIF4EBP1, TEAD4, and CCND1 participated in the WNT, PI3K, Hippo, and cell cycle pathways (Figure 2(b)). Furthermore, to validate the dysregulation of these genes in ESCC, we also collected an independent gene expression dataset to investigate their differential expression levels between ESCC and adjacent normal tissues. Specifically, all these genes were highly upregulated in ESCC as compared to the normal tissues (*P* < 0.05, Figure S1). These results indicated that the integrative analysis of the CNAs and gene expression data could identify the candidate driver genes and oncogenic pathways involved in ESCC.

### 3.3. The Consequences of CNA-Related Driver Genes on the Downstream Pathways

**Figure 3:**
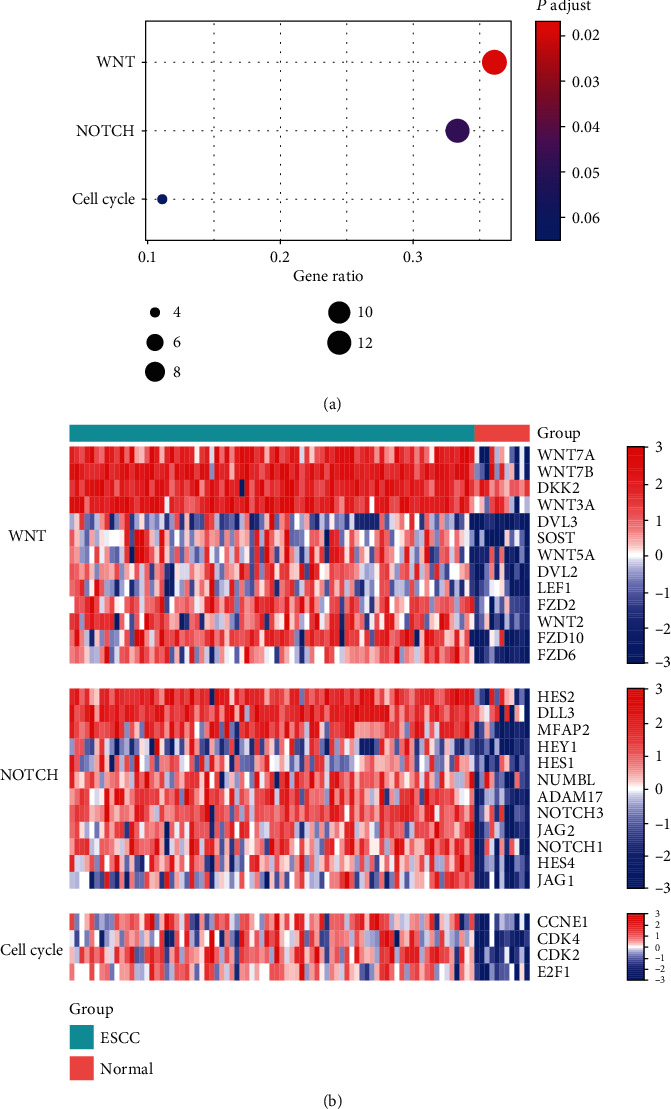
The consequences of the driver genes on the oncogenic pathways. (a) The oncogenic pathways enriched by the upregulated and downregulated genes. The node size and color represent the number of genes and statistical significance. (b) The dysregulated genes in the oncogenic pathways enriched by the differentially expressed genes.

To further investigate the consequences of the CNA-related driver genes on the downstream pathways, we conducted overrepresentation enrichment analysis (ORA) to identify the oncogenic pathways that the DEGs were enriched in. Notably, WNT, NOTCH, and cell cycle were significantly upregulated in the ESCC samples (Figure 3(a)). The ligands, including WNT7A, WNT7B, WNT3A, WNT5A, and WNT2; the receptors, including FZD2, FZD6, and FZD10; and the transcription factor, LEF1, in the WNT signaling pathway, were significantly upregulated in ESCC (Figure 3(b)). Similarly, the components in the NOTCH signaling pathway and cell cycle were also significantly elevated in ESCC compared with the adjacent normal tissues (Figure 3(b)). Collectively, the abnormal upregulation of driver genes including FZD6 and CCND1 might result in activation of the WNT and cell cycle pathways.

### 3.4. The Key Transcription Factors of Driver Gene-Mediated Oncogenic Pathways

**Figure 4:**
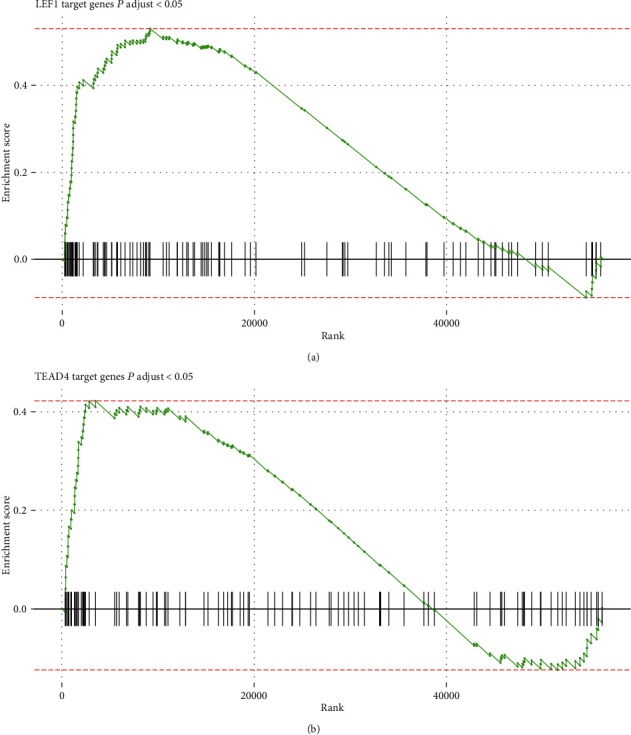
The enrichment degree of the LEF1 and TEAD4 target genes. The enrichment degree of the LEF1 and TEAD4 target genes is displayed in (a) and (b), respectively. The upregulated and downregulated target genes are represented by the black lines on the left and right, respectively.

To further interpret and verify the functionalities of the CNA-related driver genes, we investigated whether the transcriptional regulatory activities of the transcription factors (TFs) were also activated in the oncogenic pathways. Among the TFs in the oncogenic pathways, the expression levels of LEF1 and TEAD4 were found to be significantly altered in ESCC samples and they participated in the abnormally activated oncogenic pathways such as WNT and Hippo signaling. Consistently, from the gene set enrichment analysis (GSEA), we observed that the target genes of LEF1 and TEAD4 were also significantly enriched by the upregulated genes in ESCC samples (Figures 4(a) and 4(b)). The abnormal activities of the downstream target genes of LEF1 and TEAD4 further suggested that the WNT and Hippo signaling pathways were abnormally activated by the driver genes FZD6 and TEAD4, respectively.

### 3.5. The Clinical Significance of the CNA-Related Driver Genes in ESCC

**Figure 5:**
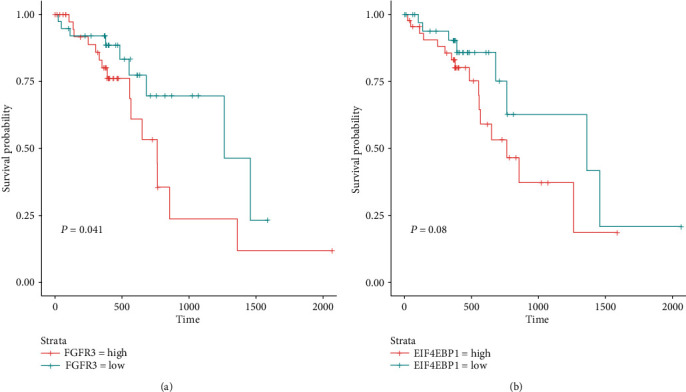
The Kaplan-Meier (KM) curves of the samples with high and low expression of driver genes. The probabilities of the samples with high and low expression of FGFR3 (a) and EIF4EBP1 (b), which are termed as high and low. The statistical significance was tested by the log-rank test.

To further explore the clinical significance of identified driver genes, we conducted survival analysis to identify those associated with ESCC overall survival. Among the six driver genes, FGFR3 and EIF4EBP1 were observed to be significantly associated with shorter overall survival in ESCC (Figures 5(a) and 5(b), *P* < 0.1, Figure S2). Moreover, ESCC samples with higher expression of the two oncogenes might have a worse prognosis than those with lower expression. These results suggested that FGFR3 and EIF4EBP1 might be unfavorable indicators in ESCC.

## 4. Discussion

Esophageal squamous cell carcinoma (ESCC) is one of the most common malignancies worldwide [17]. ESCC is mostly diagnosed at an advanced stage due to late diagnosis, leading to poor prognoses in ESCC patients [18]. However, the molecular mechanisms of the poor prognosis in ESCC are still unclear. In this study, we aimed to identify and interpret the driver genes triggered by the copy number alterations (CNAs). Specifically, we identified six driver genes including CCND1, TEAD4, EIF4EBP1, EGFR, FGFR3, and FZD6, which were highly upregulated in the samples with gains of the corresponding genes (*P* < 0.05). In the validation dataset, the six candidate driver genes were also upregulated in tumor tissues. These driver genes were involved in the oncogenic pathways including RTK-RAS, WNT, PI3K, Hippo, and cell cycle [7, 19]. Particularly, EGFR, FGFR3, and FZD6 were receptors of the signaling transduction pathways such as the RTK-RAS and WNT pathways. The CCND1 involved in cell cycle progression; TEAD4, a transcription factor in the Hippo signaling pathway; and EIF4EBP1, the translational regulator in PI3K/Akt/mTOR signaling, have been found to be significantly deregulated in several cancers [20–22]. In ESCC, CCND1 was associated with high proliferation [23], and EIF4EBP1 was an adverse prognostic indicator [24, 25]. Furthermore, to verify the consequences of the CNA-related driver genes, we examined the activities of the downstream pathways and transcription factors (TFs). Notably, the genes involved in WNT, NOTCH, and cell cycle were significantly upregulated in the ESCC samples. In addition, the target genes of TFs such as LEF1 and TEAD4 were found to be differentially expressed in ESCC samples. Based on the ORA and GSEA, we found that the upregulated genes were significantly enriched in the components of the oncogenic pathways and the target genes of LEF1 and TEAD4, respectively, suggesting that the WNT, NOTCH, cell cycle, and Hippo signaling pathways were abnormally activated by their corresponding driver genes [26, 27]. These oncogenic signaling pathways have been recurrently reported to lead to uncontrolled proliferation of cancer cells and unfavorable prognostic outcomes in several cancer types [28]. Remarkably, the transcription factor LEF1 has been found to enhance beta-catenin translocation to the nucleus in ESCC [29]. TEAD4 acted as a key regulator of the functional module in ESCC by bioinformatic analysis [30]. The ligands, receptors, and TFs transported to the nucleus in the WNT signaling pathway played key roles in the tumorigenesis and progression of ESCC [31, 32]. In addition, we observed that upregulation of FGFR3 was significantly associated with shorter overall survival in ESCC. Enhanced FGFR3 expression promotes high proliferation and causes poor prognosis in ESCC [33, 34]. Hereby, we speculated that the CNAs in the six driver genes might enhance their expression levels; activate the downstream oncogenic signaling pathways such as WNT, NOTCH, cell cycle, and Hippo via regulating their TFs such as LEF1 and TEAD4, as well as TF target genes; and consequently result in poor prognosis.

Collectively, the driver genes triggered by CNAs not only exhibited critical functionality but also were clinically relevant in ESCC, which greatly improved our understanding of the molecular mechanisms in ESCC.

## Data Availability

The raw data can be accessed from TCGA.

## Supplementary Materials

Supplementary Materials Figure S1: the expression levels of the six candidate driver genes in ESCC and normal tissues of an independent dataset. The red and grey boxes represent the ESCC and normal tissues, respectively. The mRNA intensities were used as the expression levels. Figure S2: the Kaplan-Meier (KM) curves of the samples with high and low expression of FZD1, FGFR, TEAD4, and CCND1. The red and green curves represent the groups of patients with high and low gene expression.
