# Supplementary figures and images for: Identification and Characterization of Copy Number-Associated Driver Genes in Esophageal Squamous Cell Carcinoma
Source: Biomed Res Int. 2020 Aug 22;2020:6387519. doi: 10.1155/2020/6387519 (PMC7463369; doi:10.1155/2020/6387519)

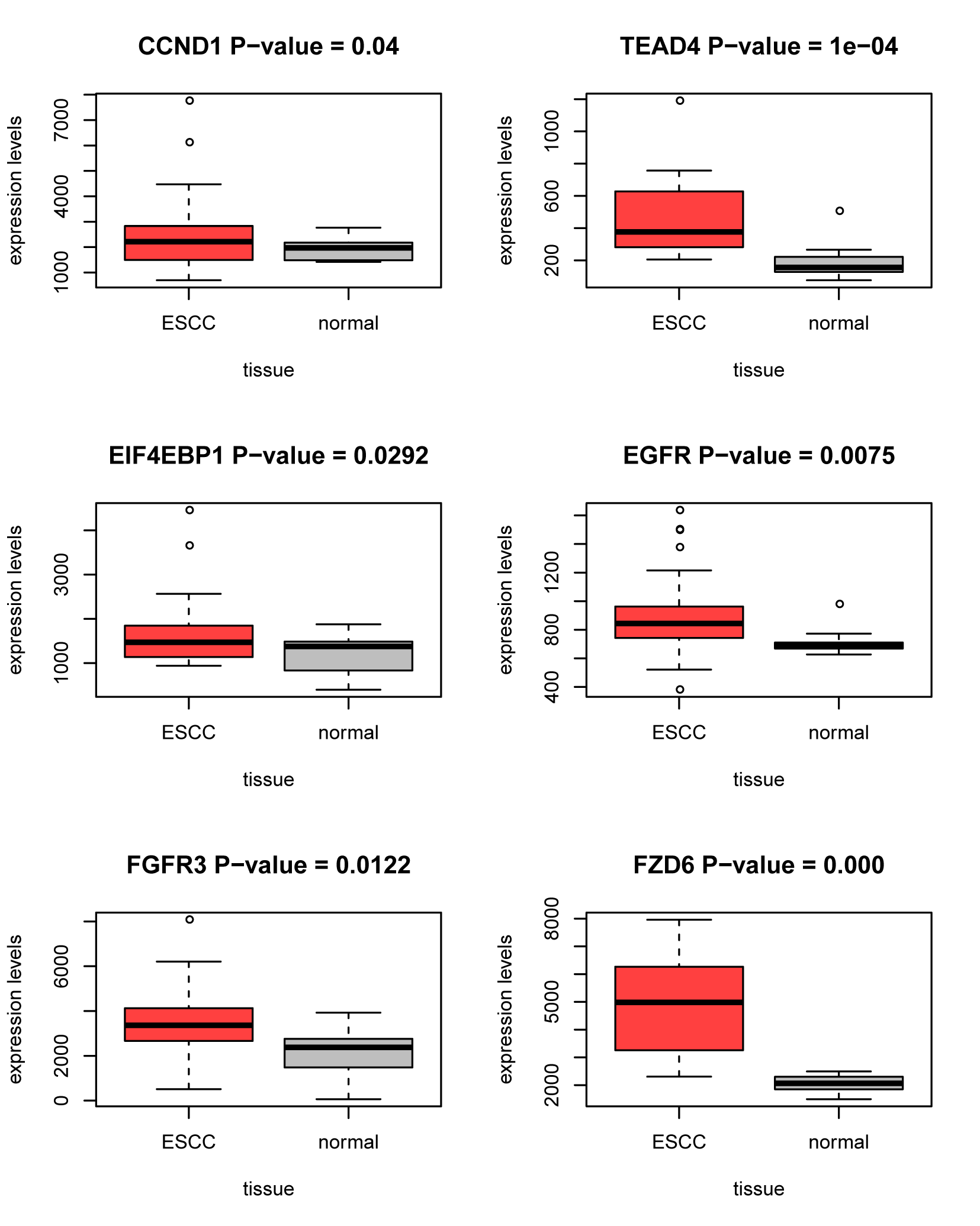

Supplement: Supplementary Materials — Figure S1: the expression levels of the six candidate driver genes in ESCC and normal tissues of an independent dataset. The red and grey boxes represent the ESCC and normal tissues, respectively. The mRNA intensities were used as the expression levels. Figure S2: the Kaplan-Meier (KM) curves of the samples with high and low expression of FZD1, FGFR, TEAD4, and CCND1. The red and green curves represent the groups of patients with high and low gene expression. [file 6387519.f1.zip › Figure S1-01.tif]

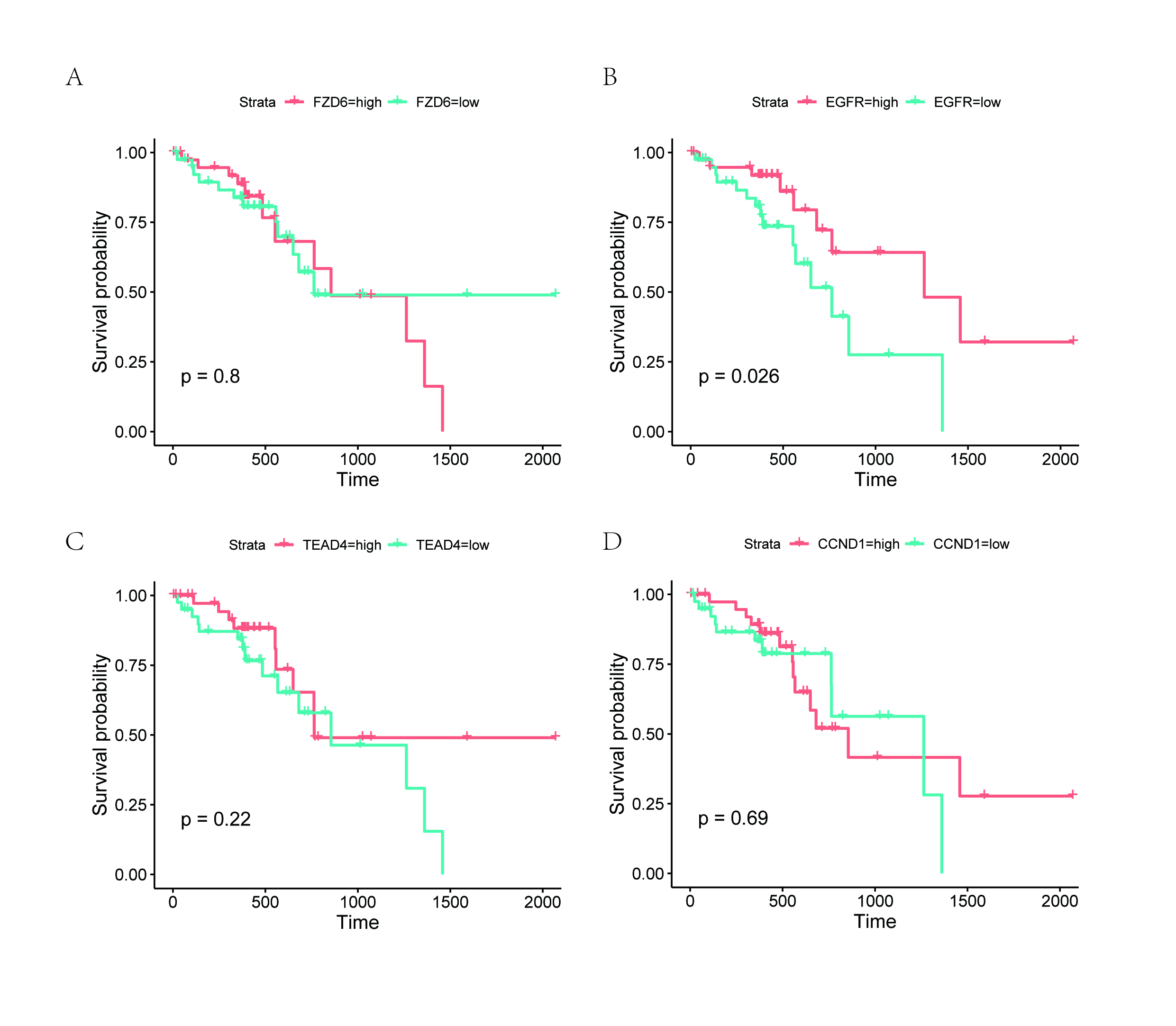

Supplement: Supplementary Materials — Figure S1: the expression levels of the six candidate driver genes in ESCC and normal tissues of an independent dataset. The red and grey boxes represent the ESCC and normal tissues, respectively. The mRNA intensities were used as the expression levels. Figure S2: the Kaplan-Meier (KM) curves of the samples with high and low expression of FZD1, FGFR, TEAD4, and CCND1. The red and green curves represent the groups of patients with high and low gene expression. [file 6387519.f1.zip › Figure S2-01.tif]
